# Trigonal multivalent polonium monolayers with intrinsic quantum spin Hall effects

**DOI:** 10.1038/s41598-022-06242-3

**Published:** 2022-02-08

**Authors:** Hairui Bao, Bao Zhao, Jiayong Zhang, Yang Xue, Hao Huan, Guanyi Gao, Zhongqin Yang

**Affiliations:** 1 State Key Laboratory of Surface Physics and Key Laboratory of Computational Physical Sciences (MOE) and Department of Physics, Fudan University, Shanghai, 200433 China; 2 School of Physics Science and Information Technology, Shandong Key Laboratory of Optical Communication Science and Technology, Liaocheng University, Liaocheng, 252059 China; 3 Jiangsu Key Laboratory of Micro and Nano Heat Fluid Flow Technology and Energy Application, School of Physical Science and Technology, Suzhou University of Science and Technology, Suzhou, 215009 China; 4 School of Science, East China University of Science and Technology, Shanghai, 200237 China; 5 Shanghai Qi Zhi Institute, Shanghai, 200030 China

**Keywords:** Topological insulators, Electronic properties and materials

## Abstract

Two-dimensional (2D) topological insulators, a type of the extraordinary quantum electronic states, have attracted considerable interest due to their unique electronic properties and promising potential applications. Recently, the successful fabrication of 2D Te monolayers (*i.e.* tellurene) in experiments (Zhu et al., Phys Rev Lett 119:106101, 2017) has promoted researches on the group-VI monolayer materials. With first-principles calculations and tight-binding (TB) method, we investigate the structures and electronic states of 2D polonium (poloniumene), in which Po is a congener of Te. The poloniumene is found to have the tendency of forming a three-atomic-layer 1 T-MoS_2_-like structure (called trigonal poloniumene), namely, the central-layer Po atoms behave metal-like, while the two-outer-layer Po atoms are semiconductor-like. This unique multivalent behavior of the Po atoms is conducive to the structural stability of the monolayer, which is found to be an intrinsic quantum spin Hall insulator with a large band gap. The nontrivial topology originates from the $p_{x,y} - p_{z}$ band inversion, which can be understood based on a built TB model. The poloniumene with different congener elements doped is also explored. Our results provide a thorough understanding of structures and electronic states of 2D polonium-related materials.

## Introduction

In the past decade, topological insulators (TIs) have attracted considerable interest in the field of condensed matter physics and materials science owing to their diverse physical properties and potential applications^1–6^. As new quantum electronic states, three-dimensional (3D) and two-dimensional (2D) TIs can exhibit unique electron transport behaviors due to their gapped bulk state with dissipationless metallic surface or edge states. In particular, quantum spin Hall (QSH) insulators, one type of the 2D TIs, are characterized by robust gapless helical edge states protected by time-reversal symmetry, which are suitable for applications in spintronics, optoelectronics, and thermoelectrics etc^7–9^. Although many materials have been theoretically predicted to have the QSH effect, only few systems such as HgTe/CdTe^10^ and InAs/GaSb/AlSb^11^ quantum wells, ZrTe_5_^12,13^, and bismuthene^14^ have been confirmed in experiments. It is thus of great significance to explore new materials with the QSH effect to enrich the 2D TI families. At present, some effective methods, including atom doping^15,16^, chemically functionalization^17,18^, electric field^19^, strain^20^, and substrates effects^21^, have been employed to obtain the QSH materials. Compared to the 2D TIs generated by these external strategies, intrinsic 2D TIs are more desirable due to their numerous advantages, such as the experimental feasibility and the absence of scattering from the extra atoms etc.

Since the discovery of graphene, the 2D mono-elemental materials (such as group-III^22^, group-IV^23^, and group-V^14^ monolayers (MLs)) have been extensively investigated. With the successful fabrication of the 2D tellurene in tetragonal structure in experiments^24^, the realm of 2D materials has been extended to the group-VI elements^25–27^. As a congener element with Te in group VI, Po MLs have also been recently studied and predicted to form a square lattice as the ground state^27^. The material is, however, unstable without the consideration of spin–orbit coupling (SOC) due to the Peierls instability^27,28^. Although polonium is radiative, polonium and polonium related materials have actually attracted considerable attention in physical, chemical, and materials science due to their rich and interesting properties and promising applications^29–32^. The structural phases, densities of states, and electronic band structures of a simple cubic polonium have been studied based on first principles calculations^29–31^.

In this work, we explore electronic states of the poloniumene with a different structure based on first-principles calculations and tight-binding (TB) model. We find that poloniumene tends to form the 1 T-MoS_2_-like structure (called trigonal poloniumene). This structure is more stable than the square-lattice ground state proposed in Ref.^27^. The trigonal poloniumene is a semiconductor without SOC. It changes to a QSH insulator with a large band-gap (0.29 eV) when the SOC is turned on. The structure is stable even before the SOC is considered, unlike the square poloniumene. The nontrivial topology originates from the $p_{x,y} - p_{z}$ band inversion, which can be explained by a TB model. It is interesting to note that the obtained topological state is robust against a wide range of biaxial strain (− 5% ~ 5%). It can also be well maintained even if the poloniumene is on a hexagonal BN (*h*-BN) substrate or under an external electric field. The structural, electronic, and topological properties of the trigonal poloniumene with different congener elements doped are also explored.

## Results and discussion

### Geometric structures and stability

**Figure 1:**
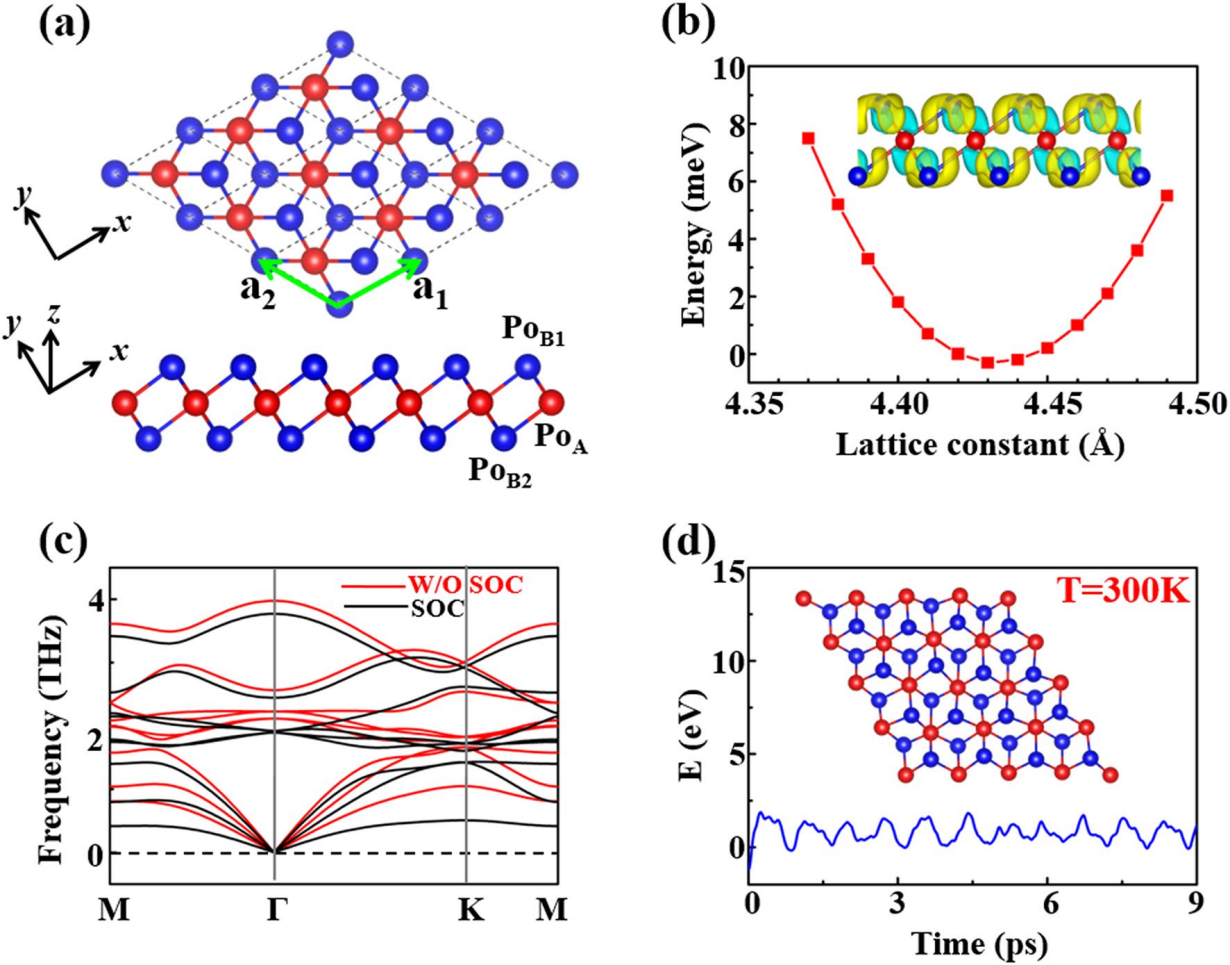
(**a**) Top and side views of the trigonal poloniumene. The red (blue) spheres denote the Po atoms at the central (outer) layer(s). (**b**) The total energy as a function of the lattice constant, where the total energy (− 8.918 eV) of the equilibrium structure with *a*_0_ = 4.43 Ǻ is set as zero energy reference. The inset shows the difference of the charge densities between the poloniumene and the isolated Po atoms. The yellow color indicates gaining electrons, while the blue indicates losing electrons. (**c**) Phonon dispersion curves of the trigonal poloniumene without (red curves) and with (black curves) SOC. (**d**) Energy variation of the trigonal poloniumene during the AIMD simulations at 300 K, where the total energy (− 140 eV) is set as zero energy reference. The inset displays a snapshot of the final frame of the poloniumene at a time of 9 ps.

The crystal structure we propose for the poloniumene is 1 T-MoS_2_-like structure (Fig. 1a), with space group *P-*3*m*1 and point group D_3d_. This structure is different from the square lattice that is brought into focus in Ref.^27^ and one of the structures proposed for the tellurene in Ref.^24^. The primitive unit cell of the trigonal poloniumene contains three Po atoms, namely the central-layer Po_A_ as well as the outer-layer Po_B1_ and Po_B2_ atoms, as indicated by the red and blue spheres in Fig. 1a. Each central-layer Po_A_ is neighbored with six outer-layer Po_B_ while each outer-layer Po_B_ is neighbored with three central-layer Po_A_ due to the C_3_ rotation symmetry. The optimized lattice constant of the trigonal poloniumene is 4.43 Å (Fig. 1b) and the calculated bond length between Po atoms is 3.20 Å. The obtained cohesive energy of 3.25 eV/atom shows strong bonding in the trilayer 1 T-MoS_2_-like poloniumene, which is larger than that of the square poloniumene^27^ by 0.033 eV/atom. Thus, we find a more stable structure than the square lattice for the poloniumene and the square lattice is not the ground state for the 2D Po ML.

According to the Bader charge analysis, the charge transfer from the central-layer atom to the up (low)-layer atom is 0.21 e/atom in the trigonal poloniumene. The trend of the charge transfer can also be seen clearly from the distribution of the charge density difference in the material (the inset of Fig. 1b), indicating the multivalent behaviors of the Po atoms. Specifically, the central-layer Po atom tends to be metal-like while the outer-layer Po atoms prefer the semiconductor-like feature, just like the bonding in MoS_2_ structure^33^. This unique multivalent behavior of the Po atoms makes the structure very stable. The dynamic stability of the trigonal poloniumene is confirmed by the phonon spectra (Fig. 1c). No negative frequencies appear in the calculated phonon spectra without and with SOC of the trigonal poloniumene, in contrast to the square poloniumene^27^. To demonstrate the thermal stability of the pristine trigonal poloniumene, ab initio molecular dynamics (AIMD) simulations with a 4 × 4 × 1 supercell are performed. The AIMD result at the temperature of 300 K is displayed in Fig. 1d. The poloniumene keeps the stable configuration after being heated for more than 9 ps at 300 K, implying the thermal stability of the trigonal poloniumene at room temperature.

### Band structures and intrinsic QSH effects

**Figure 2:**
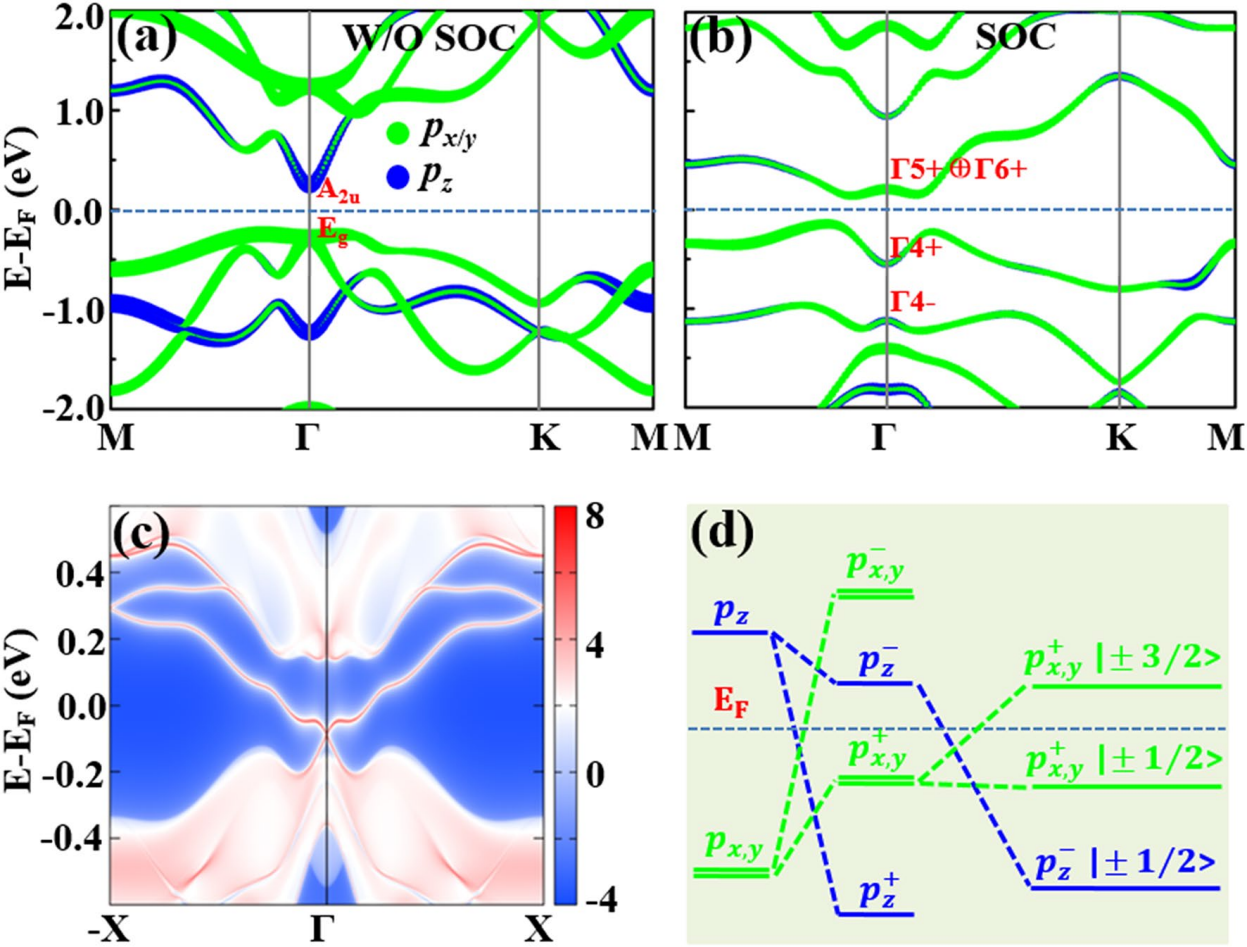
Orbital-resolved band structures of the poloniumene without (**a**) and with (**b**) SOC, where the calculated irreducible representations of the energy bands around the E_F_ are also displayed. The label + ( −) denotes the even (odd) parity. (**c**) Helical edge states of the poloniumene. (**d**) Schematic diagram of the energy level evolution for the $p_{x,y}$ and $p_{z}$ orbitals around the Γ point.

The electronic band structures without and with SOC of the trigonal poloniumene are shown in Figs. 2a, b, in which E_F_ indicates the Fermi level. In the absence of SOC, the poloniumene is the normal insulator with a band gap of 0.49 eV, smaller than that of the Te ML^24^, due to the enhanced metallic behavior of Po atoms. Its conduction band minimum is just located at Γ point while the valence band maximum deviates slightly from the Γ point (Fig. 2a). As shown in Fig. 2a, the orbitals at the conduction band minimum and valence band maximum are mainly the $p_{z}$ and $p_{x,y}$ states, respectively. Moreover, the concerned $p_{z}$ and $p_{x,y}$ states (Fig. S1 in the Supplementary Information) are contributed mainly by the central-layer Po_A_ and the outer-layer Po_B1_ and Po_B2_ atoms, respectively. In the presence of SOC, a large band gap of 0.29 eV is obtained for the poloniumene.

To identify the topology of the band structure (Fig. 2b), we calculate the *Z*_2_ invariant based on parities^34^ for the material since the ML has inversion symmetry. The *Z*_2_ invariant is determined by the parities of the occupied state wave functions at the time-reversal invariant momentum points in the Brillouin zone (BZ) as $\left( { - 1} \right)^{\nu } = \mathop \prod \limits_{i} \delta_{i}$ with $\delta_{i} = \mathop \prod \limits_{m = 1}^{N} \xi_{2m} \left( {{\Gamma }_{i} } \right)$ (without SOC), where the $\xi = \pm 1$ is the parity eigenvalues of the wave functions at the four time-reversal invariant momentum points (*i.e.*, one Γ point and three M points), $N$ is the number of occupied states, and $\nu$ represents the *Z*_2_ index. The parity values of each band at the four time-reversal invariant momentum points can be obtained from the first-principles calculations. The topological index $\nu = 1$ ($\nu = 0$) corresponds to the topologically nontrivial (trivial) states of the system. For the trigonal poloniumene, the parities of the energy levels at ${\Gamma }$ are marked in Fig. 2a, b (only partly occupied bands are shown). In the presence of SOC, the calculated parity products for the occupied states at the time-reversal invariant points Γ and Μ are -1 and 1, respectively. Hence, the nontrivial topological invariant $\nu = 1$ is obtained, indicating an intrinsic QSH insulator with a large band gap for the poloniumene.

As illustrated clearly in Fig. 2a, b, the strong SOC of the heavy Po atoms induces the band inversion between $p_{z}$ and $p_{x,y}$ orbitals even if the band gap in Fig. 2a is as large as about 0.5 eV. This tendency can also be seen by the irreducible representations shown in Fig. 2a, b. The doubly degenerate E_g_ bands in Fig. 2a are split into two bands (Γ4 + , Γ5 +  ⊕ Γ6 +) by the SOC. And the A_2u_ (odd parity) band in Fig. 2a turns to the Γ4- band in Fig. 2b, lower in energy than the bands of Γ4 + and Γ5 +  ⊕ Γ6 + . This band inversion drives the trigonal poloniumene into the topologically nontrivial QSH state. In contrast, the Te ML with the 1 T-MoS_2_-like structure is a trivial insulator^24^ since the SOC of Te atom is not large enough to invert the energy bands (about 1.05 eV at Γ) but only decreases the band gap. The achieved topologically nontrivial band gaps (0.29 eV for the indirect band gap and 0.76 eV for the direct band gap at Γ) are large enough to observe the QSH effect at room temperature in the ML. This magnitude is significantly larger than those of many previously reported intrinsic topological insulators, such as 23.9 meV in germanene^23^ and 8.6 meV in organometallic lattices^35^. Therefore, the trigonal poloniumene is an intrinsic topological insulator with a very large nontrivial band gap.

**Figure 3:**
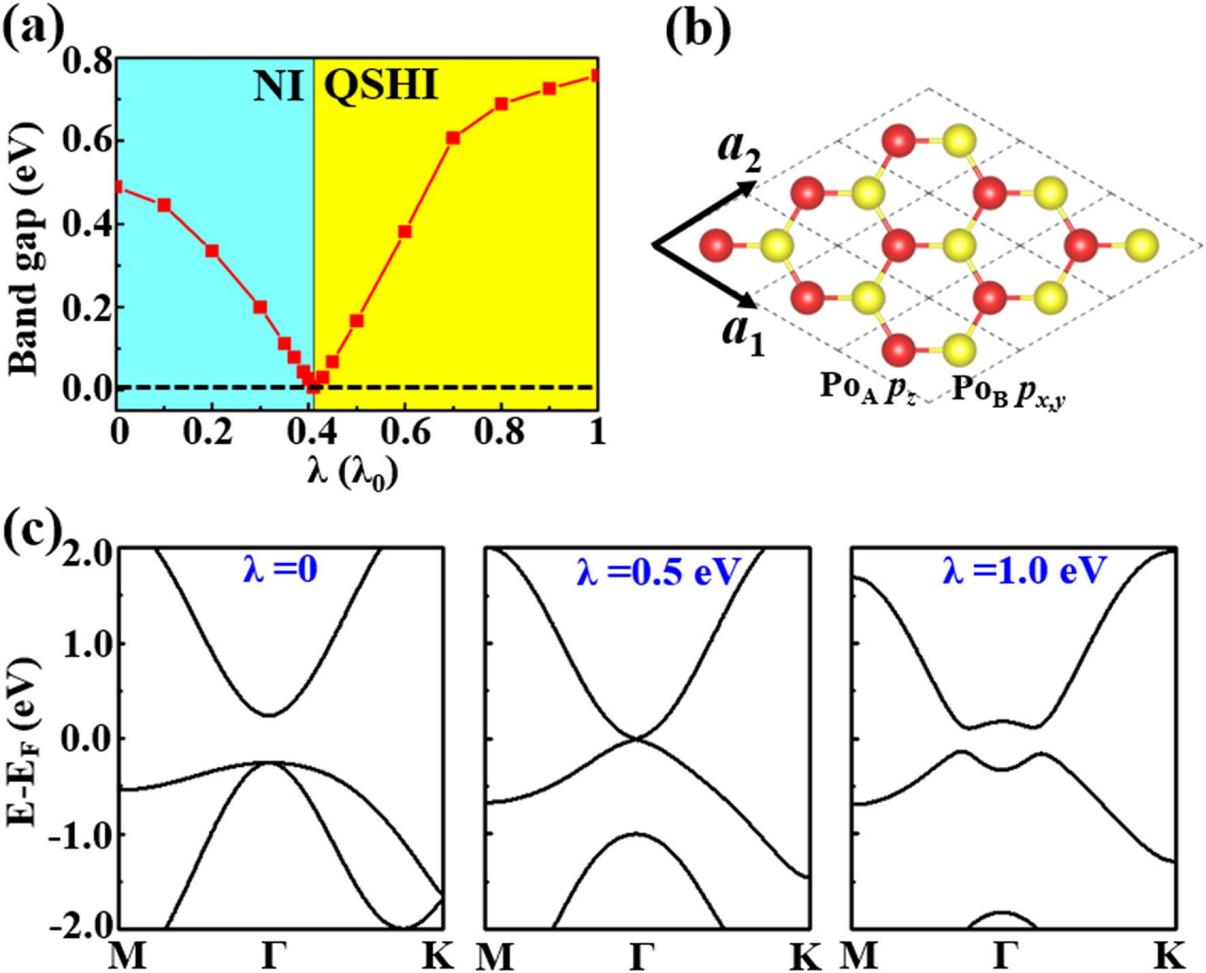
(**a**) Direct band gaps at Γ point and topological phases versus the SOC magnitude. The ‘NI’ and ‘QSHI’ mean the normal and QSH insulators, respectively. (**b**) Schematic plot of a bitriangular lattice with Po_A_ $p_{z}$ and Po_B_ $p_{x,y}$ orbitals. (c) Band structures from the TB model based on the bitriangular lattice with $\lambda$ = 0.0, 0.5, and 1.0 eV.

To deeply explore the topologically nontrivial properties, the edge states of the poloniumene are calculated. The obtained edge states of a semi-finite trigonal poloniumene are illustrated in Fig. 2c. A helical edge states connecting the conduction and valence bands appear explicitly inside the bulk gap, with a Fermi velocity of about 0.8 $\times 10^{6} { }m \cdot s^{ - 1}$, comparable to that ($1 \times 10^{6} { }m \cdot s^{ - 1}$) of graphene^36^. Figure 2d demonstrates the origin of nontrivial topology. At the beginning, the $p$ orbital of the Po atom splits into degenerate $p_{x,y}$ and nondegenerate $p_{z}$ orbitals due to the C_3_ symmetry of the 1 T-MoS_2_-like lattice. Due to the bonding between the neighboring Po atoms, these states near the E_F_ can split into the bonding and anti-bonding states, *i.e.*, ($p_{x,y}^{ + }$,$p_{z}^{ + }$) and ($p_{x,y}^{ - }$, $p_{z}^{ - }$), respectively, in which signs + and − denote the parities. After the SOC is turned on, the band inversion between $p_{z}^{ - }$ and $p_{x,y}^{ + }$ states around the E_F_ occurs, explaining well the topological mechanism of the system. How the SOC affects the topological electronic state is also investigated. Figure 3a gives the nontrivial band gap of the poloniumene as a function of the SOC strength λ = 0 ~ λ_0,_ where λ_0_ is the real SOC strength of the ML. With the increase of the λ, the band gap first decreases, then closes (at about 0.41 λ_0_), and finally reopens. Thus, a topological phase transition from a normal insulator to a QSH insulator occurs when the band gap closes (about 0.41 λ_0_), in accordance with the band inversion mechanism discussed above. According to the parity analysis, the *Z*_2_ invariant is determined simply by the parities of the highest occupied bands at Γ point. As expected, the parity flips at 0.41 λ_0_, leading to $\nu = 1$ for λ > 0.41 λ_0_ and the QSH state.

### Tight-binding model

As discussed above the nontrivial topology originates from the band inversion from the $p_{z}$(around the bottom of the conduction bands) and $p_{x,y}$(around the top of the valence bands) states, which can be further comprehended based on a TB model. According to the atom-resolved band structures (Fig. S1), the $p_{z}$ state at the bottom of the conduction bands mainly comes from the Po_A_ atoms, while the $p_{x,y}$ state at the top of the valence bands primarily comes from the Po_B1_ and Po_B2_ atoms. Since the $p_{x,y}$ distributions of the Po_B1_ and Po_B2_ atoms are completely the same (Fig. S1), they can be regarded as one unit. Thus, a simplified bi-triangular lattice^37^ is built for the TB model (Fig. 3b). On the basis of $\left\{ {p_{z}^{A} , p_{x}^{B} , p_{y}^{B} } \right\}$, the model Hamiltonian can be expressed as

1 $$H = \left( {\begin{array}{*{20}c} {H_{zz}^{A} } & {H_{zx}^{AB} } & {H_{zy}^{AB} } \\ {H_{zx}^{*AB} } & {H_{xx}^{B} } & {H_{xy}^{B} } \\ {H_{zy}^{*AB} } & {H_{xy}^{*B} } & {H_{yy}^{B} } \\ \end{array} } \right) + \left( {\begin{array}{*{20}c} 0 & 0 & 0 \\ 0 & 0 & { - i\lambda } \\ 0 & {i\lambda } & 0 \\ \end{array} } \right),$$

with the matrix elements of

$$\begin{aligned} H_{zz}^{A} & = \varepsilon_{z} + V_{pp\pi AA} \left[ {4\cos \left( {\frac{\sqrt 3 }{2}k_{x} } \right)\cos \left( {\frac{1}{2}k_{y} } \right) + 2{\text{cos}}\left( {k_{y} } \right)} \right], \\ H_{xx}^{B} & = \varepsilon_{xy} + \left( {3V_{pp\sigma BB} + V_{pp\pi BB} } \right){\text{cos}}\left( {\frac{\sqrt 3 }{2}k_{x} } \right)\cos \left( {\frac{1}{2}k_{y} } \right) + 2V_{pp\pi BB} {\text{cos}}\left( {k_{y} } \right), \\ H_{yy}^{B} & = \varepsilon_{xy} + \left( {V_{pp\sigma BB} + 3V_{pp\pi BB} } \right){\text{cos}}\left( {\frac{\sqrt 3 }{2}k_{x} } \right)\cos \left( {\frac{1}{2}k_{y} } \right) + 2V_{pp\sigma BB} {\text{cos}}\left( {k_{y} } \right), \\ H_{zx}^{AB} & = {\text{D}}\left( {V_{pp\sigma AB} - V_{pp\pi AB} } \right)\left[ {e^{{i\frac{\sqrt 3 }{3}k_{x} }} - e^{{ - i\frac{\sqrt 3 }{6}k_{x} }} \cos \left( {\frac{1}{2}k_{y} } \right)} \right], \\ H_{zy}^{AB} & = i\sqrt 3 {\text{D}}\left( {V_{pp\sigma AB} - V_{pp\pi AB} } \right)\left[ {\sin \left( {\frac{1}{2}k_{y} } \right)e^{{ - i\frac{\sqrt 3 }{6}k_{x} }} } \right], \\ H_{xy}^{B} & = \sqrt 3 \left( {V_{pp\pi BB} - V_{pp\sigma BB} } \right){\text{sin}}\left( {\frac{\sqrt 3 }{2}k_{x} } \right)\sin \left( {\frac{1}{2}k_{y} } \right). \\ \end{aligned}$$

In the above formulae, the $\varepsilon_{z}$ and $\varepsilon_{xy}$ are on-site energies of the Po_A_ $p_{z}$ and Po_B_ $p_{xy}$ orbitals, respectively, $V_{pp\pi AA}$, $V_{pp\sigma BB}$, $V_{pp\pi BB}$, $V_{pp\sigma AB}$, and $V_{pp\pi AB}$ are hopping parameters, $D = \sin \theta \cos \theta$ with ${\uptheta }$ being the angle between the *z*-axis and bonding, which can be fixed as $\pi /3$ in the calculations^16^. Figures S2a, b show the fitted band structures without and with SOC from the TB model, respectively. One finds that the band structures around the E_F_ obtained from the TB Hamiltonian are consistent with the density functional theory (DFT) results and aptly illustrate the nontrivial topology. The fitted parameters are $\varepsilon_{z} = 1.710$ eV, $\varepsilon_{xy} = - 1.170$ eV, $V_{pp\pi AA} = - 0.245$ eV, $V_{pp\sigma BB} = 0.387$ eV, $V_{pp\pi BB} = - 0.081$ eV, $V_{pp\sigma AB} = - 0.100$ eV, $V_{pp\pi AB} = 0.530$ eV and $\lambda = 1.000$ eV. The band inversion mechanism as a function of the SOC magnitude can be explained well by using the TB model based on the bitriangular lattice. As shown in Fig. 3c, the band gap is closed in the TB model with λ = 0.5 eV, indicating the topological transition from a normal insulator to a topological insulator.

### Tuning the electronic states by substitutional doping

**Figure 4:**
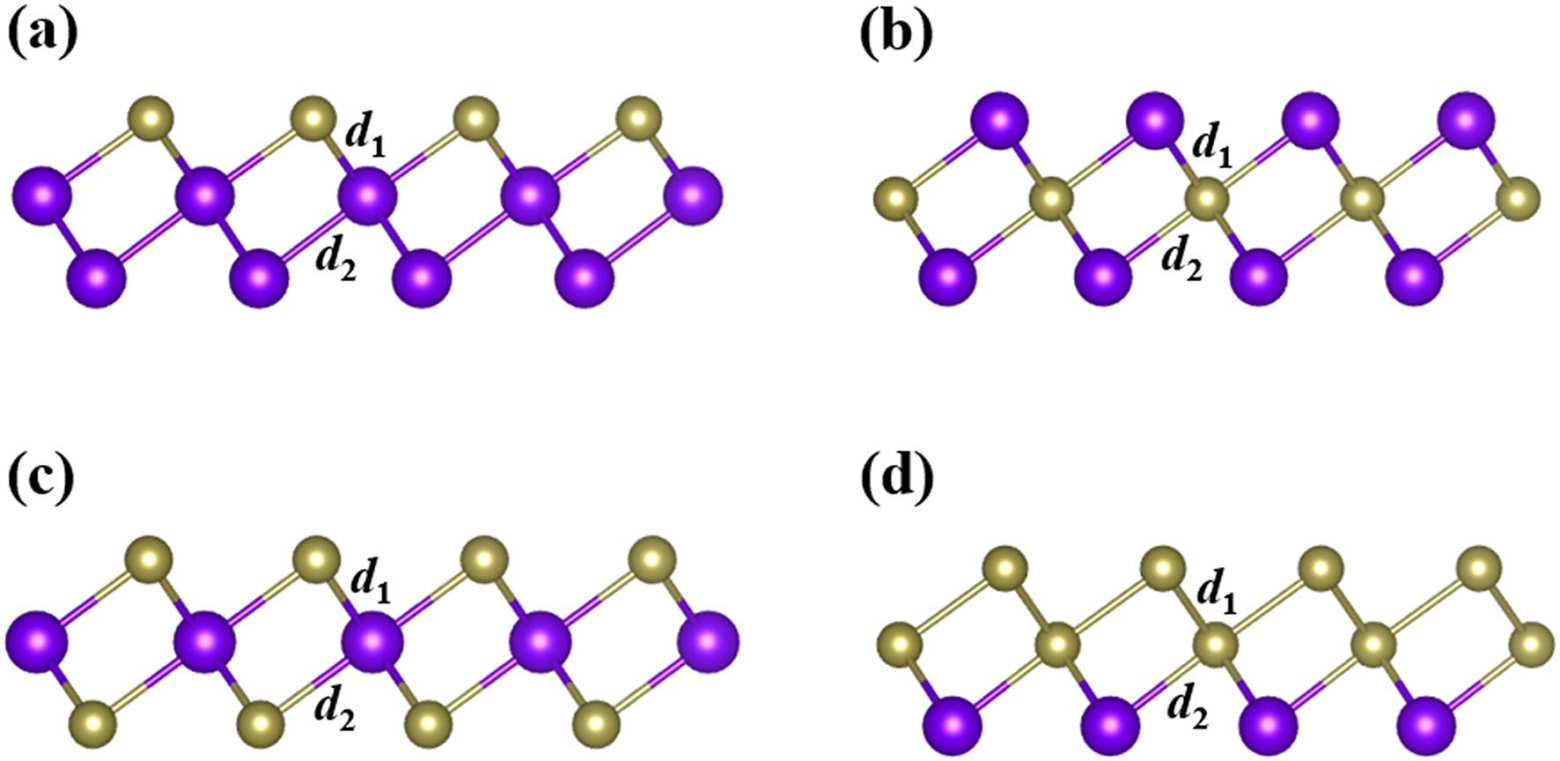
Geometrical structures for the trigonal poloniumene MLs with four doping patterns of (**a**) XPoPo, (**b**) PoXPo, (**c**) XPoX, and (**d**) XXPo, in which X = S, Se, Te atoms. The purple spheres indicate the Po atoms while the brown spheres denote the X atoms.

In 2D materials, substitutional doping^38^ is an effective method of engineering the electronic band structures, which may induce many interesting physical properties and effects. The congener substitutional doping can also directly tune the SOC strength, which is very important to the appearance of the topological states. As illustrated in Fig. 4, we investigate four different patterns of (a) XPoPo, (b) PoXPo, (c) XPoX, and (d) XXPo for the poloniumene with X (X = S, Se, Te) atoms doped. The patterns XPoPo and XXPo exhibit the Janus-like structures^38^ while the patterns PoXPo and XPoX still belong to the typical 1 T-MoS_2_-like ones. Table 1 presents the related results for the trigonal poloniumene MLs with the four different patterns. As expected, the lattice constants for the four different patterns all increase gradually with X varying from S, Se to Te. To evaluate the structural stability, we calculate the corresponding cohesive energies with the formula $E_{{{\text{coh}}}} = \left( {mE_{{{\text{Po}}}} + nE_{{\text{X}}} - E_{{{\text{tol}}}} } \right)/3$, where m (n) denotes the numbers of Po (X) atom, $E_{{{\text{Po}}}}$, $E_{{\text{X}}}$, and $E_{{{\text{tol}}}}$ are the total energies of the insolated Po atom, X atom, and one unit cell of the doped trigonal poloniumene ML, respectively. The calculated cohesive energies (with SOC) for the four different patterns XPoPo, PoXPo, XPoX, and XXPo are in the range of 2.6 ~ 3.2 eV/atom. Thus, all of the doped systems have large cohesive energies, implying their structural stability. We also calculate the phonon spectra for these doped trigonal poloniumene MLs, as shown in Fig. S3. Obviously, the absence of negative frequencies for all of the Te doped systems shows that they are dynamically stable. It should be pointed out that all of the O doped poloniumene systems exhibit negative phonon spectra and are not discussed in detail.

**Table 1:** The lattice constant *a*, bond length *d*_1_ (*d*_2_) between the central-layer atom and the up-layer (low-layer) atom, charge transfer ΔQ1 (ΔQ2) from the central-layer atom to the up-layer (low-layer) atom, cohensive energy *E*_coh_, band gap *E*_g_, electronic states (including normal insulating (NI), metallic (M), and TI), and dynamic stability for the trigonal poloniumene ML with X (X = S, Se, Te) atoms doped. The SOC is considered.

Pattern	Atom	*a*(Å)	*d*_1_(Å)	*d*_2_(Å)	Δ*Q*1	Δ*Q*2	*E*_coh_(eV/atom)	*E*_g_(eV)	State	Stable
XPoPo	S	4.21	2.815	3.221	0.64	0.16	3.172	0.37	TI	Y
	Se	4.28	2.945	3.231	0.49	0.16	3.074	0.30	TI	Y
	Te	4.41	3.152	3.245	0.29	0.16	2.989	0.19	TI	Y
PoXPo	S	3.92	2.954	2.954	− 0.30	− 0.31	3.028	0.00	M	N
	Se	4.11	3.036	3.036	− 0.16	− 0.16	2.975	0.00	TI	Y
	Te	4.36	3.185	3.185	0.02	0.02	2.970	0.37	TI	Y
XPoX	S	3.96	2.778	2.778	0.65	0.65	3.185	0.63	NI	Y
	Se	4.11	2.918	2.918	0.50	0.50	2.954	0.46	NI	Y
	Te	4.36	3.135	3.135	0.30	0.30	2.750	0.16	NI	Y
XXPo	S	3.75	2.495	2.839	0.18	− 0.28	2.721	0.19	NI	N
	Se	3.98	2.722	2.969	0.18	− 0.14	2.689	0.07	NI	N
	Te	4.32	3.071	3.163	0.17	0.05	2.693	0.03	TI	Y

The Bader charge analysis is performed for all of the doped systems. According to the obtained results, the electrons of the central-layer atoms tend to transfer to the up-layer or the low-layer atoms, which is consistent with the metal-like and semiconductor-like behaviors for the central-layer atoms and outer-layer atoms, respectively, as in the trigonal poloniumene. Take pattern XPoX as an example, the transferred electron numbers diminish from S, Se to Te due to the gradually decreasing electronegativity. For patterns PoXPo and XPoX, the charge transfer ΔQ1 is equal to ΔQ2 because of the inversion symmetry owned in the structures. For the Janus-like structures XPoPo and XXPo, the charge transfer ΔQ1 is reasonably different from the ΔQ2. Thus, the signs and values of ΔQ1 and ΔQ2 can be well comprehended based on the electronegativities of the elements and the metal-like (semiconductor-like) behaviors of the atoms in the central (outer) layers.

**Figure 5:**
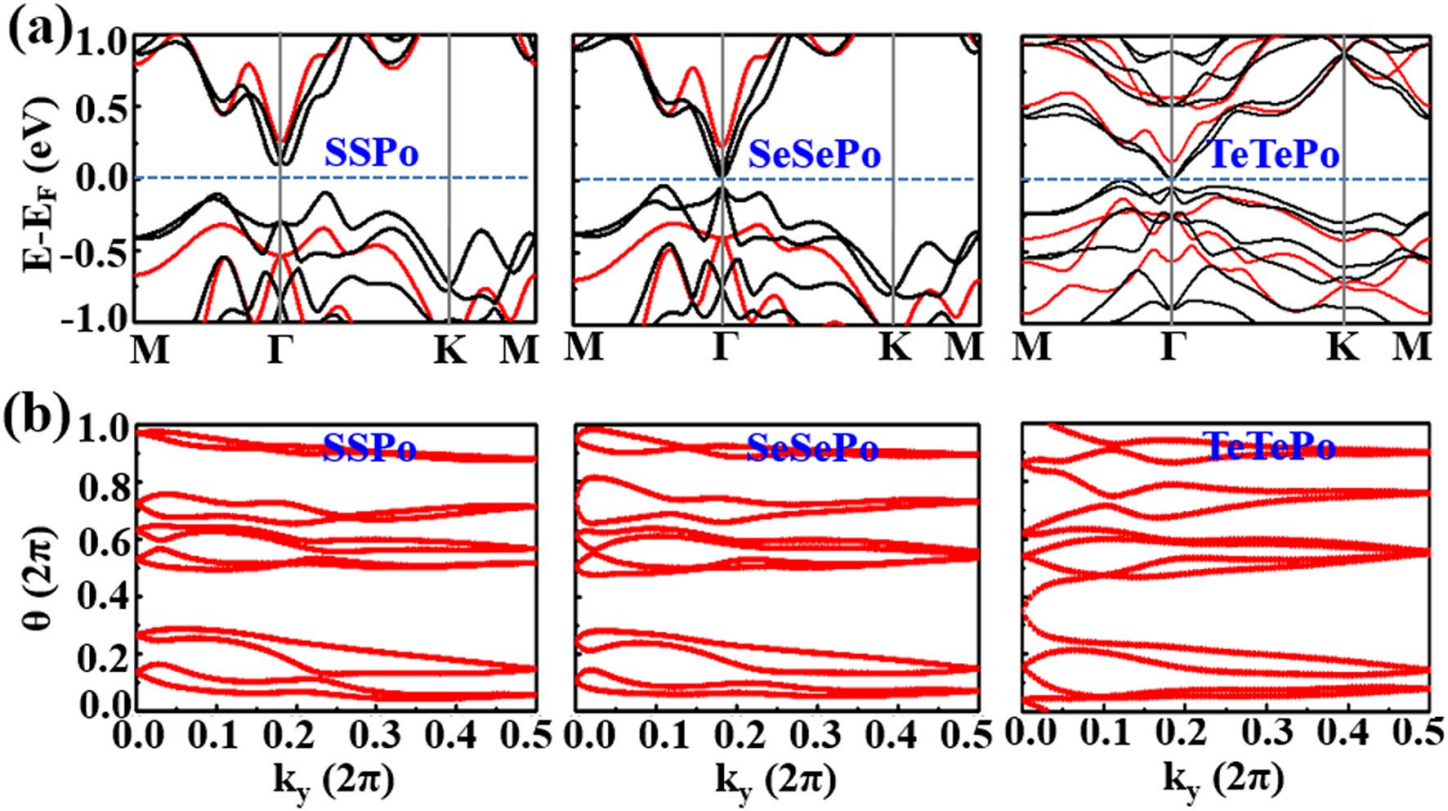
(**a**) Band structures of the XXPo (X = S, Se, Te) MLs without (red curves) and with (black curves) SOC. (**b**) gives the corresponding Wannier charge centers.

After the SOC is considered, obvious Rashba effects^39^ appear in the band structures (Fig. S4) of the Janus-like doped systems of XPoPo and XXPo due to the structure inversion asymmetry. It is reasonable that the Rashba effect in SPoPo is the largest. As displayed in Table 1, except PoSPo, all other PoXPo and all XPoPo are topological insulators due to the low X doping concentration. Interestingly, relatively large band gaps are obtained in the XPoX materials without SOC (Fig. S4), which is more analogous to the 1 T-MoS_2_ than the other three patterns. Since the band inversion cannot be triggered by the SOC, only normal insulators are achieved for all of the XPoX materials (Table 1). For XXPo, the MLs have closely sizable band gaps in the absence of SOC (Fig. 5a). After the SOC is taken into account, the band gap of the XXPo ML decreases with X varying from S, Se to Te. Particularly, a phase transition from normal insulators (SSPo and SeSePo) to a topological insulator (TeTePo) happens in the process, confirming by the evolution of the Wannier charge centers (Fig. 5b). The above analysis indicates that the substitutional doping can not only alter the values of the band gaps but also induce the phase transitions between the topological insulators and normal insulators or metals.

### Effects of strain, external electric fields, and substrates

**Figure 6:**
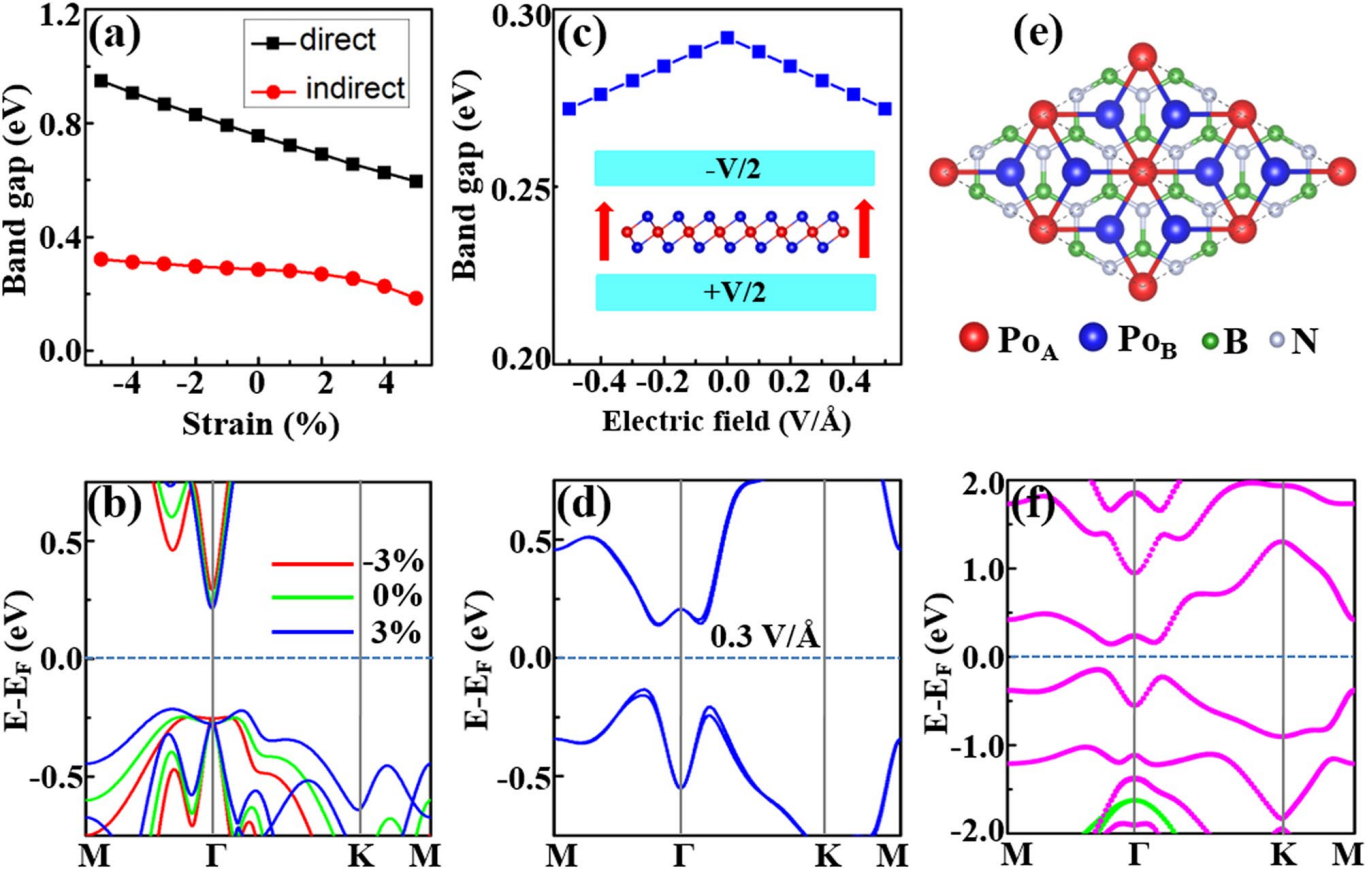
(**a**) Direct and indirect band gaps of the trigonal poloniumene as a function of the in-plane biaxial strain. (**b**) Band structures without SOC under the different strains. (**c**) Global band gaps of the trigonal poloniumene as a function of the external electric field. The inset shows the schematic diagram of the material with an external electric field. (**d**) Band structure of the poloniumene with SOC under the electric field of 0.3 V/Å. (**e**) Top view of the trigonal poloniumene on an *h*-BN substrate. (**f**) Band structure of the trigonal poloniumene/*h*-BN heterostructure with SOC. The magenta and green bands are contributed by the states from the trigonal poloniumene and the *h*-BN substrate, respectively.

We now explore the effects of in-plane biaxial strain on the nontrivial topology of the trigonal poloniumene. The in-plane biaxial strain is defined as $\varepsilon = \left( {a - a_{0} } \right)/a_{0}$, where $a$ $(a_{0} )$ is the strained (unstrained) lattice constant. Note that $\varepsilon > 0$ ($\varepsilon < 0$) corresponds to the biaxial tensile (compressive) strain. Figure 6a shows the direct band gap at Γ point and global indirect band gap of the poloniumene as a function of biaxial strain. Interestingly, the two types of the band gaps both remain open in the range of biaxial strain of − 5% ~ 5%. Thus, the topological index does not change and no topological phase transitions appear during the process, indicating the robustness of the band topology of the system. The indirect band gap slightly changes with the strain in the range of -5% ~ 5%. On the contrary, the direct band gap at Γ point presents a near-linear variation with the strain (Fig. 6a). For the strain varying from − 5 to 5%, the in-plane lattice constant increases while the ML thickness decreases (from 4.05 ${\AA}$ to 3.61 ${\AA}$), leading to the decrease of the coupling interaction between $p_{x,y}$ and $p_{z}$. Thus, the energy split between $p_{x,y}$ and $p_{z}$ around the E_F_ at Γ point as shown in Fig. 6b decreases. Since the charge transfer between the central-layer Po and the outer-layer Po decreases, the in-plane potential gradient decreases, which causes the decrease of the SOC of the $p_{x,y}$ orbitals and thus the decrease of the topologically nontrivial direct band gap during the process.

For many 2D materials, the electronic band structures can also be effectively modulated by applying an external electric field. As shown in Fig. 6c, we study the global band-gap variation of the trigonal poloniumene under the external electric field in the range of − 0.5 ~ 0.5 ${\text{V}}/{\AA}$. The calculated band structure with SOC in the presence of 0.3 ${\text{V}}/{\AA}$ is illustrated in Fig. 6d. Compared to Fig. 2b, obvious Rashba splitting^39,40^ is induced by the electric field in the bands of Fig. 6d. The magnitude of the Rashba splitting increases with the electric field, leading to the linear decrease of the band gap in Fig. 6c with the increase of the electric field from zero to 0.5 ${\text{ V}}/{\AA}$. Due to the inversion symmetry of the poloniumene, the band-gap variation is symmetric upon the flipping of the electric-field direction (Fig. 6c). Since the band gap remains open under the external electric field in the range of − 0.5 ~ 0.5 ${\text{V}}/{\AA}$, it could be said that the nontrivial topology of the trigonal poloniumene is robust against external electric fields.

We also explore suitable substrate materials for the poloniumene. As displayed in Fig. 6e, the poloniumene can be deposited on an *h*-BN ML, which has been synthesized experimentally and owns a wide bulk gap^41^. The lattice constant of the 1 × 1 trigonal poloniumene ML is very close to that of the $\sqrt 3 \times \sqrt 3$ supercell of the *h*-BN ML and the lattice mismatch between them is less than 1%. As show in Fig. S5, three patterns (*i. e.*, T_1_, T_2_, and H) are considered. The H stacking configuration is found being the most stable with its total energy lower by 1.0 (1.6) meV than T_1_ (T_2_) stacking configurations. The interlayer distance between trigonal poloniumene and *h*-BN ML is about 4.34 Å, indicating that a weak van der Waals heterostructure is formed. The calculated band structures with SOC for the trigonal poloniumene/h-BN heterostructure are shown in Fig. 6f. Due to the weak interaction from the substrate, the nontrivial band structure of the trigonal poloniumene is still maintained in the heterostructure. The *h*-BN substrate has almost no influence on the bands around the E_F_ of the trigonal poloniumene. The green band in Fig. 6f denotes the contributions from *h*-BN substrate. Therefore, the *h*-BN ML is a suitable substrate for the trigonal poloniumene.

Since polonium atoms are radioactive, they may transfer to Pb, Bi, or Tl atoms during the radioactive process, we explore the electronic structures of the trigonal poloniumene with Pb, Bi, or Tl atoms doped to simulate one of the effects from the radiation and decay. As shown in Fig. S6, two typical doping patterns are considered. The doping concentration is about 8%. The QSH effect in these doping systems is identified by the calculations of the evolution of the Wannier charge centers. Take Pb atom doped poloniumene as an example, as displayed in Fig. S7. In the absence of SOC, the systems with both doping patterns exhibit trivial metallic properties. In the presence of SOC, the two systems both transform into large band-gap topological insulators (with the E_F_ moving to 0.3 eV). Similar results are obtained for the Bi and Tl cases (not shown). These obtained results indicate that the doping of Pb, Bi, or Tl atoms (decaying products) in the trigonal poloniumene does not alter the topological behavior. Some other effects, such as structural stability etc., may also exist in the radiative process, which require further investigations in the future.

## Conclusion

The structural, electronic, and topological properties of the trigonal poloniumene monolayer are investigated by the first-principles method and tight-binding model. A trilayer 1 T-MoS_2_-like structure, which is dynamically and thermally stable, is proposed for Po films. It is interesting to find that this trigonal poloniumene is an intrinsic topological insulator with a large global band gap (0.29 eV). The nontrivial topology is identified by the non-zero *Z*_2_ invariant, evolution of the Wannier charge center, and the helical edge states. The topological mechanism can be ascribed to the $p_{x,y - } p_{z}$ band inversion, which can be comprehended based on a TB model. Many stable materials with various electronic structures including the topological insulating states are achieved when the trigonal poloniumene monolayers are doped with cognate elements. Both the in-plane strain and external electric field can significantly tune the nontrivial band gap of the trigonal poloniumene. Our results provide a thorough understanding of the structural and electronic properties of the Po and its related materials.

## Methods

The geometry optimization and electronic structure calculations of the trigonal poloniumene ML are performed by the projector-augmented-wave potential^42,43^ formalism based on ab initio DFT. The Perdew-Burke-Ernzerhof generalized-gradient approximation (GGA-PBE) is employed to describe the exchange and correlation functional^44^. The DFT-D2 dispersion correction^45^ is used to describe the long-range van der Waals interactions for the system with *h*-BN substrates. The plane-wave basis with a kinetic cut-off energy of 500 eV and the convergence criterion for the total energy of 10^–6^ eV are adopted. The vacuum space is set to be larger than 20 Å to avoid the artificial interaction between the two adjacent monolayers. All atoms in the unit cell are fully relaxed until the Hellmann–Feynman force on each atom is smaller than 0.01 eV/Å. The Γ centered Monkhorst–Pack grids of 13 × 13 × 1 are adopted for the calculations. To investigate the dynamic stability of the trigonal poloniumene ML, the phonon dispersion is calculated by using the DFT perturbation theory as implemented in the PHONOPY code. The thermal stability of the optimized structures is performed by the finite-temperature AIMD simulations^46^. A TB model is built to further understand the band structures from the DFT calculations. To obtain the edge states, we construct a TB Hamiltonian with a basis of maximally localized Wannier functions^47,48^. The electric field is applied in VASP package by adding an artificial dipole sheet in the supercell^49^. The dipole correction is turned on to avoid interactions between the periodically repeated images. The direction of the applied electric field is perpendicular to the plane of the trigonal poloniumene ML (Fig. 6c), and the magnitude of the electric field varies from -0.5 V/Å to 0.5 V/Å. All of the crystal structures in this paper are drawn by VESTA package^50^.

## Supplementary Information

Supplementary Information.
