# Supplementary material for: Trigonal multivalent polonium monolayers with intrinsic quantum spin Hall effects
Source: Sci Rep. 2022 Feb 8;12:2129. doi: 10.1038/s41598-022-06242-3 (PMC8826415; doi:10.1038/s41598-022-06242-3)
Supplement: Supplementary file 1 — Supplementary Information. [file 41598_2022_6242_MOESM1_ESM.docx]

Supplementary Information

**Trigonal multivalent polonium monolayers with intrinsic quantum spin Hall effects**

Hairui Bao^1^, Bao Zhao^1,2^, Jiayong Zhang^3^, Yang Xue^4,1^, Hao Huan^1^, Guanyi Gao^1^, and Zhongqin Yang^1,5^*

*^1^State Key Laboratory of Surface Physics and Key Laboratory of Computational Physical Sciences (MOE) & Department of Physics, Fudan University, Shanghai 200433, China*

*^2^School of Physics Science and Information Technology, Shandong Key Laboratory of Optical Communication Science and Technology, Liaocheng University, Liaocheng 252059, China*

*^3^Jiangsu Key Laboratory of Micro and Nano Heat Fluid Flow Technology and Energy Application, School of Physical Science and Technology, Suzhou University of Science and Technology, Suzhou 215009, China*

*^4^School of Science, East China University of Science and Technology, Shanghai 200237, China*

*^5^Shanghai Qi Zhi Institute, Shanghai 200030, China*

**Corresponding Author**

*E-mail: [zyang@fudan.edu.cn](mailto:zyang@fudan.edu.cn)


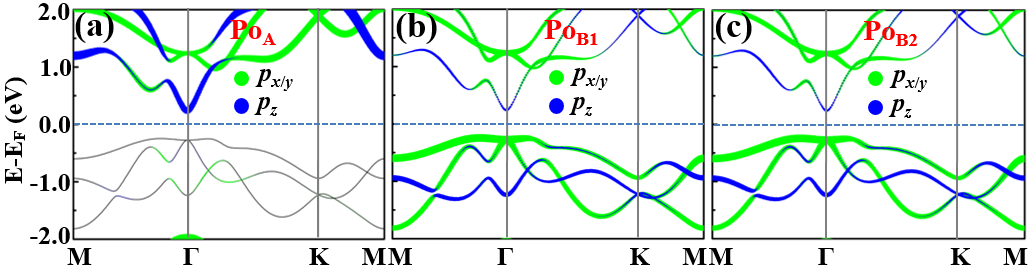


**Fig. S1** Atom-resolved band structures for (a) Po_A_, (b) Po_B1_, and (c) Po_B2_ of the trigonal poloniumene.


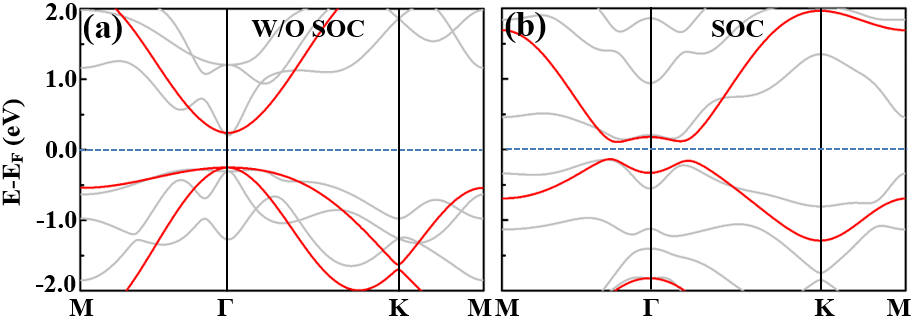


**Fig. S2** Band structures without (a) and with (b) SOC of the poloniumene, where the gray and red curves denote the DFT and TB model results, respectively.


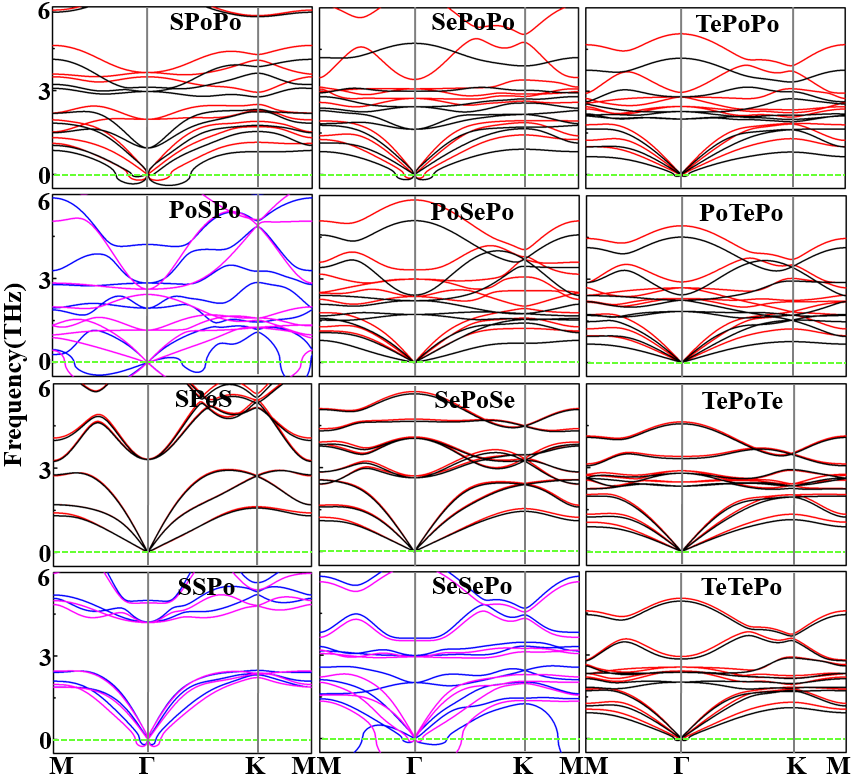


**Fig. S3** Phonon spectra of the doped trigonal poloniumene MLs with the four patterns of XPoPo, PoXPo, XPoX, and XXPo, where X = S, Se, Te. The red and black curves denote the dynamically stable systems without and with the consideration of the SOC, respectively, while the blue and magenta curves indicate the dynamically unstable systems without and with the consideration of the SOC, respectively. Note that the negative phonon dispersion exists in range of -7.5~-2.5 THz (not shown) for the SSPo ML.


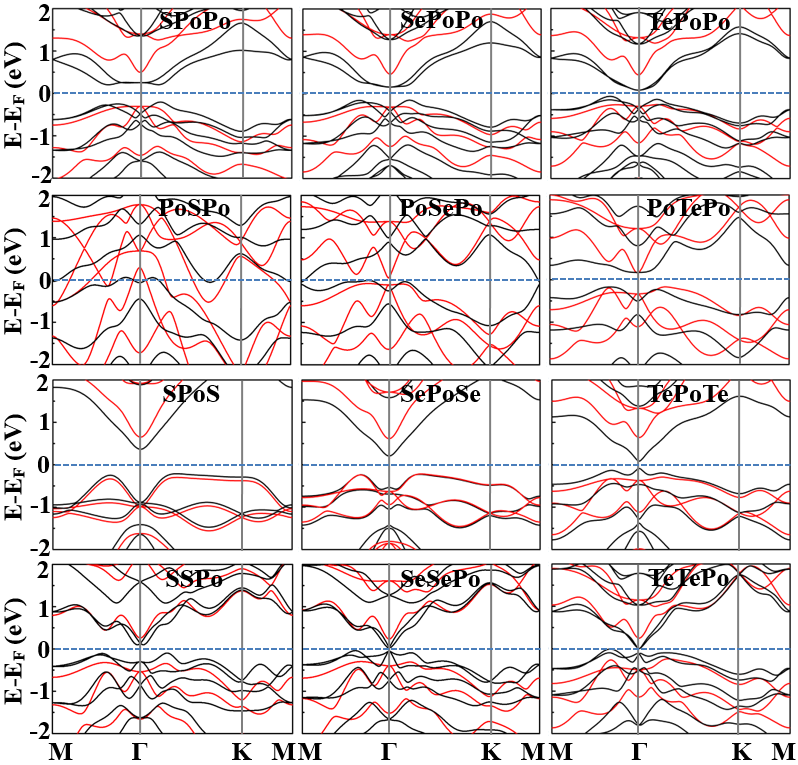


**Fig. S4** Band structures for the doped trigonal poloniumene ML without (red curves) and with (black curves) SOC.


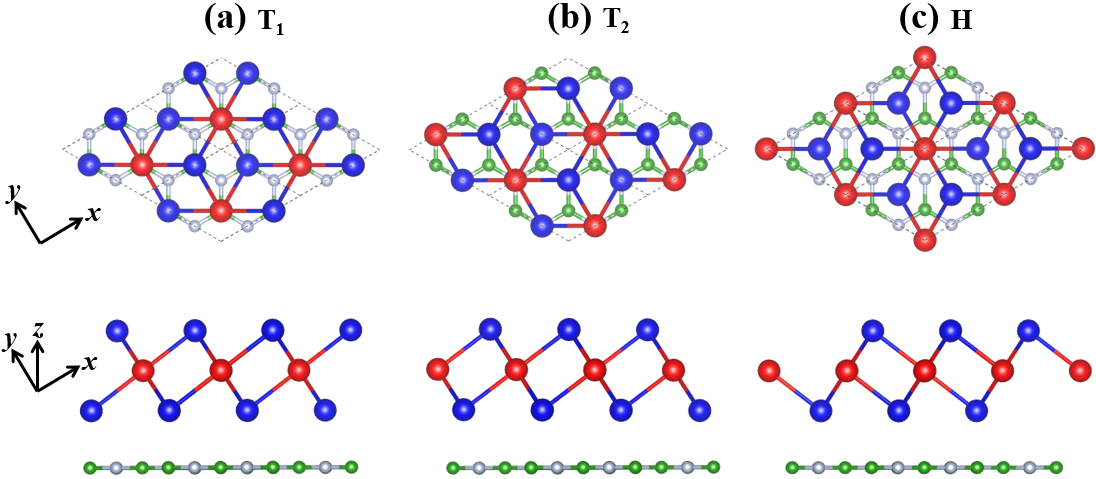


**Fig. S5** Top and side views of the trigonal poloniumene/BN heterostructure with three types of staking configurations. (a), (b), and (c) panels denote the T_1_, T_2_, and H stacking configurations, respectively.


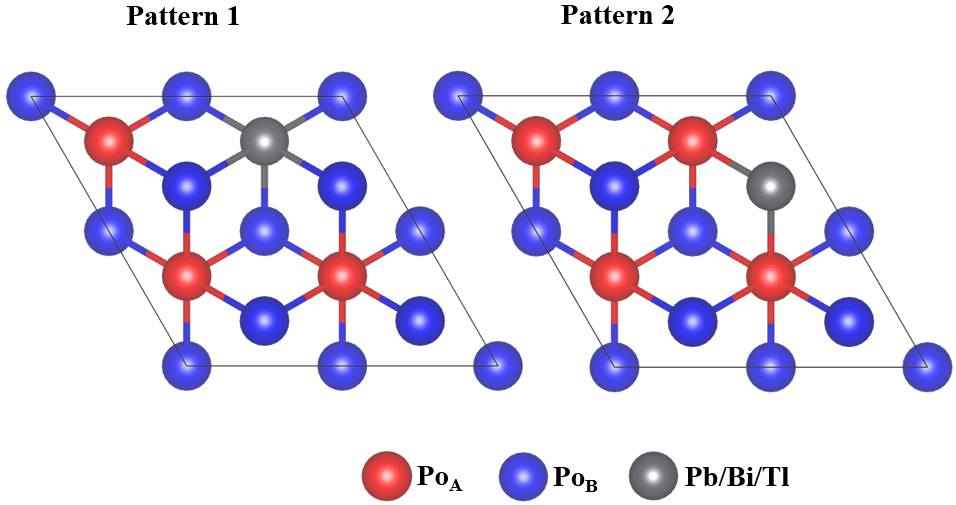


**Fig. S6** Two geometrical structures for the trigonal poloniumene MLs with Pb/Bi/Tl atoms (gray color) doped.


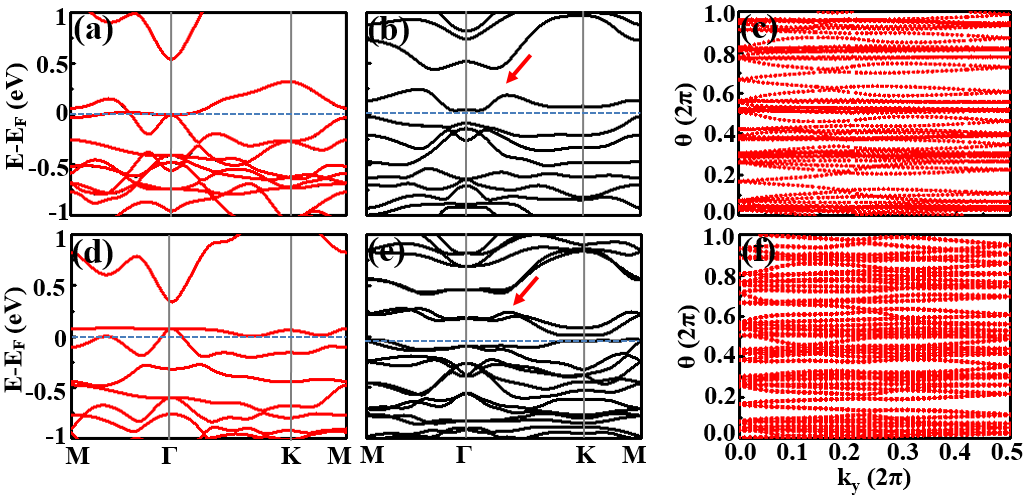


**Fig. S7** The band structures without (a) and with (b) SOC as well as Wannier charge center (c) for the poloniumene with Pb atoms doped in the pattern 1 structure. The results in (d-f) are the same as in (a-c), respectively, except for the pattern 2 structure.
